# Bacterial Adrenergic Sensors Regulate Virulence of Enteric Pathogens in the Gut

**DOI:** 10.1128/mBio.00826-16

**Published:** 2016-06-07

**Authors:** Cristiano G. Moreira, Regan Russell, Animesh Anand Mishra, Sanjeev Narayanan, Jennifer M. Ritchie, Matthew K. Waldor, Meredith M. Curtis, Sebastian E. Winter, David Weinshenker, Vanessa Sperandio

**Affiliations:** a Department of Microbiology, University of Texas Southwestern Medical Center, Dallas, Texas, USA; b Department of Biochemistry, University of Texas Southwestern Medical Center, Dallas, Texas, USA; c School of Pharmaceutical Sciences, State University of Sao Paulo (UNESP), Araraquara, Brazil; d Department of Diagnostic Medicine/Pathobiology, Kansas State University, Manhattan, Kansas, USA; e Department of Microbial Sciences, University of Surrey, Guildford, United Kingdom; f Brigham and Women’s Hospital, Howard Hughes Medical Institute, Division of Infectious Diseases, Harvard Medical School, Boston, Massachusetts, USA; g Department of Human Genetics, Emory University School of Medicine, Atlanta, Georgia, USA

## Abstract

Enteric pathogens such as enterohemorrhagic *Escherichia coli* (EHEC) and *Citrobacter rodentium*, which is largely used as a surrogate EHEC model for murine infections, are exposed to several host neurotransmitters in the gut. An important chemical exchange within the gut involves the neurotransmitters epinephrine and/or norepinephrine, extensively reported to increase virulence gene expression in EHEC, acting through two bacterial adrenergic sensors: QseC and QseE. However, EHEC is unable to establish itself and cause its hallmark lesions, attaching and effacing (AE) lesions, on murine enterocytes. To address the role of these neurotransmitters during enteric infection, we employed *C. rodentium*. Both EHEC and *C. rodentium* harbor the locus of enterocyte effacement (LEE) that is necessary for AE lesion formation. Here we show that expression of the LEE, as well as that of other virulence genes in *C. rodentium*, is also activated by epinephrine and/or norepinephrine. Both QseC and QseE are required for LEE gene activation in *C. rodentium*, and the *qseC* and *qseE* mutants are attenuated for murine infection. *C. rodentium* has a decreased ability to colonize dopamine β-hydroxylase knockout (Dbh^−/−^) mice, which do not produce epinephrine and norepinephrine. Both adrenergic sensors are required for *C. rodentium* to sense these neurotransmitters and activate the LEE genes during infection. These data indicate that epinephrine and norepinephrine are sensed by bacterial adrenergic receptors during enteric infection to promote activation of their virulence repertoire. This is the first report of the role of these neurotransmitters during mammalian gastrointestinal (GI) infection by a noninvasive pathogen.

## IMPORTANCE

The epinephrine and norepinephrine neurotransmitters play important roles in gut physiology and motility. Of note, epinephrine and norepinephrine play a central role in stress responses in mammals, and stress has profound effects on GI function. Bacterial enteric pathogens exploit these neurotransmitters as signals to coordinate the regulation of their virulence genes. The bacterial QseC and QseE adrenergic sensors are at the center of this regulatory cascade. *C. rodentium* is a noninvasive murine pathogen with a colonization mechanism similar to that of EHEC, enabling the investigation of host signals in mice. The presence of these neurotransmitters in the gut is necessary for *C. rodentium* to fully activate its virulence program, in a QseC/QseE-dependent manner, to successfully colonize its murine host. Our study data provide the first example of epinephrine and norepinephrine signaling within the gut to stimulate infection by a bacterial pathogen in a natural animal infection.

## INTRODUCTION

The survival of an organism is dependent on its intrinsic ability to detect and efficiently respond to stress cues. The neurotransmitters epinephrine (Epi) and norepinephrine (NE) play a central role in stress responses in mammals. Notably, stress affects gastrointestinal (GI) function, leading to increased gastric acid production and intestinal motility, and can also alter the composition of the gut microbiota (1). Both epinephrine and norepinephrine have important biological roles in the human GI tract. Norepinephrine is synthesized locally within the enteric nervous system (ENS) by adrenergic neurons in the basal-lateral layer of the gut (2). Epinephrine is mostly synthesized in the adrenal medulla but can reach the gut through the bloodstream (3). These neurotransmitters play important GI functions, modulating intestinal smooth muscle contraction, submucosal blood flow, and chloride and potassium secretion (4). There is an important relationship between the gut microbiota and the availability of active epinephrine and/or norepinephrine in the lumen. These neurotransmitters are inactivated by the host by glucuronidation, and the GI microbiota encodes glucuronidases that deconjugate glucuronic acid from epinephrine and norepinephrine, increasing the levels of these biologically active neurotransmitters in the lumen (5).

Moreover, epinephrine and/or norepinephrine have direct effects on bacterial physiology and virulence gene expression through interaction with the bacterial adrenergic receptors QseC and QseE (see Fig. 3A) (6–24). The role of epinephrine and/or norepinephrine in stimulating virulence gene expression has been extensively studied in the human enteric pathogen enterohemorrhagic *Escherichia coli* (EHEC) (11, 19–21, 23, 25, 26). EHEC is a foodborne pathogen responsible for major outbreaks of bloody diarrhea and hemolytic uremic syndrome (HUS) worldwide (27). EHEC colonizes the colon, where it forms attaching and effacing (AE) lesions on enterocytes. The locus of enterocyte effacement (LEE) pathogenicity island contains most of the genes necessary for AE lesion formation. The LEE contains 41 genes, the majority of which are organized within five major operons: *LEE1* to *LEE5* (28–30). The LEE genes encode a type III secretion system (T3SS) (31), an adhesin (intimin) (32) and its receptor (Tir) (33), and transcriptional regulators, chaperones, and effector proteins (34–38). EHEC senses the host neurotransmitters epinephrine and/or norepinephrine through QseC and QseE, thereby relaying notification of the presence of these chemical signals to a complex regulatory cascade and leading to transcription of key virulence genes. QseC is at the top of this signaling cascade, and, upon sensing epinephrine, it activates expression of the *qseEF* genes. In addition to genetic regulation between these two systems, there is also cross talk at the phosphorylation level. QseC phosphorylates three response regulators (RRs), its cognate RR QseB, KdpE, and QseF. QseE, however, exclusively phosphorylates QseF. This signaling cascade operating via QseC directly activates transcription of the LEE through KdpE. LEE transcriptional expression is indirectly controlled through QseEF. QseEF repress expression of *rcsB*, which encodes an RR that also activates LEE transcription. Hence, the increased expression of *rcsB* in a *qseE* mutant leads to increased LEE expression (11, 19–21, 23–26). These transcription events ultimately enable the organism to form AE lesions and produce Shiga toxin (responsible for HUS), thereby leading to the clinical manifestations of infection (7, 19, 20, 23).

However, studies of the role of these neurotransmitters in EHEC virulence during mammalian infection have been lacking because EHEC does not form AE lesions on the intestine of mice, and studies of the EHEC-host relationship at the level of intestinal disease have had to rely on expensive and genetically intractable animal models. A variety of other animal species, including mice, gnotobiotic piglets, baboons, ferrets, and calves, have been used as models to study the virulence of EHEC (39–48). EHEC is able to colonize streptomycin-treated mice; however, it does not cause AE lesions in these animals and its toxicity in this model is solely due to Shiga toxin (44, 45), given that these results could be reproduced using an *E. coli* K-12 strain carrying cloned *stx* genes (encoding Shiga toxin) (45). Ferrets (streptomycin treated) were also evaluated as a possible small-animal model for EHEC infection (46). These animals developed hematuria and /or histological damage of glomeruli or thombocytopenia, but there was no evidence of colitis or AE lesion formation in intestinal epithelial cells. Thus, the ferret and the streptomycin-treated mouse models may serve as models for renal disease secondary to intestinal infection with EHEC. EHEC is able to cause AE lesions in large animals such as suckling neonatal piglets (42) and neonatal calves (39). However, these animals are expensive and difficult to manage for screening large numbers of potential virulence genes. Finally, the nonhuman primate animal models (baboons) are limited by the high cost. In the infant rabbit animal model, EHEC colonizes the large intestine, forming AE lesions and causing diarrhea (49, 50), but, unlike murine models, rabbits are genetically intractable, hampering studies assessing the host genetic contribution to EHEC intestinal infection. An alternative used by many investigators in the field is to study *Citrobacter rodentium* as a surrogate for EHEC. This natural murine pathogen, like EHEC, harbors the LEE (see Fig. 3B) and forms AE lesions on the intestine of mice, leading to colonic hyperplasia (51, 52). All of the known virulence genes of EHEC have been validated *in vivo* using *C. rodentium* murine infections (52–56). The utilization of the *C. rodentium* model capitalizes on merging the powerful genetically tractability of host and pathogen to unravel the mechanisms involved in host recognition and infection*.* Here we employed EHEC and *C. rodentium* animal models to investigate the role of epinephrine/norepinephrine and the bacterial adrenergic receptors QseC and QseE in the pathogenesis of noninvasive enteric pathogens.

## RESULTS

### The role of QseC and QseE in mammalian infections.

**FIG 1:**
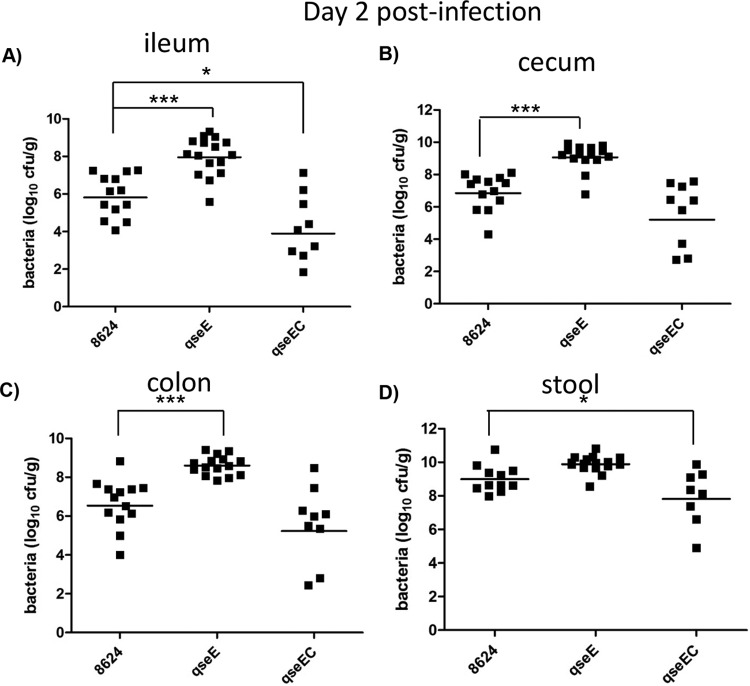
EHEC infection in infant rabbits at day 2 postinfection. Data depict CFUs of EHEC in the ileum (A), cecum (B), colon (C), and stool (D). *, *P* < 0.05; ***, *P* < 0.001.

**FIG 2:**
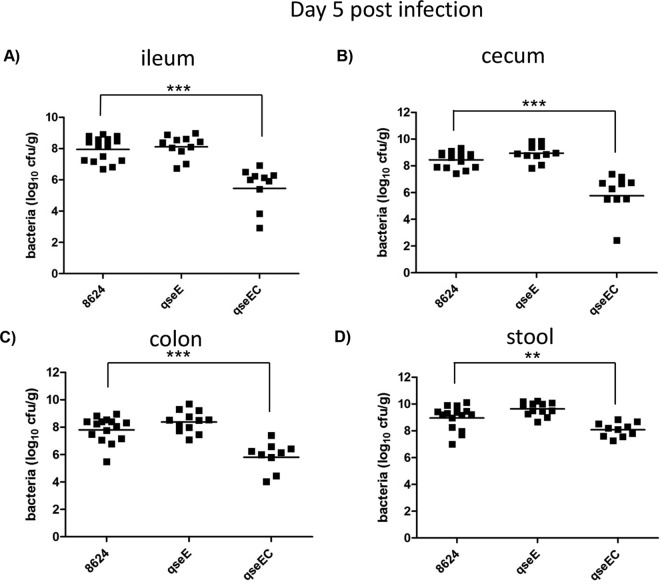
EHEC infection in infant rabbits at day 5 postinfection. Data depict CFUs of EHEC in the ileum (A), cecum (B), colon (C), and stool (D). **, *P* < 0.01; ***, *P* < 0.001.

The most suitable small-animal model for EHEC is the infant rabbit model, where EHEC colonizes the colon of these animals, forming AE lesions and causing disease. The proficiency of EHEC to colonize the intestine of these mice correlates with the severity of disease; hence, the assessment of CFUs of EHEC in the intestine of these animals provides a numeric readout of disease (49, 50). Using this infection model, we have previously shown that an EHEC *qseC* mutant is attenuated, with a decreased ability to colonize the colon, ileum, and cecum of these animals on day 5 (D5) postinfection (7). However, we did not know the contribution of the QseE adrenergic sensor for EHEC infection of infant rabbits. Moreover, we also have not investigated the pathogenicity of a double-sensor mutant. We have now assessed pathogenesis of EHEC *qseE* and *qseEC* mutants using this model. The *qseE* mutant initially (day 2 postinfection) colonizes the ileum and colon of these animals to higher levels than the wild type (WT) (Fig. 1) and later during infection (day 5) behaves similarly to the WT (Fig. 2). This initial higher colonization by the *qseE* mutant is congruent with the overexpression of the LEE genes (24). We have previously reported that, although *qseC* and *qseE* individual mutants can still sense epinephrine and norepinephrine to regulate certain arms of this signaling cascade, an EHEC *qseEC* mutant is unable to sense these neurotransmitters (24). The EHEC *qseEC* mutant presents decreased colonization of the ileum on day 2 postinfection (Fig. 1) and decreased colonization of the ileum, cecum, and colon and less bacteria within the stool at day 5 compared to the WT (Fig. 2), having a phenotype that mirrors that of the EHEC *qseC* mutant during infection of infant rabbits (7). This is not a surprising result, because QseC activates expression of the *qseEF* genes, being at the top of this signaling cascade (57). These data suggest that the ability to sense epinephrine and norepinephrine through both the QseC and QseE sensors promotes EHEC’s virulence during mammalian infection.

### Epinephrine and norepinephrine regulate virulence gene expression in *C. rodentium*.

**FIG 3:**
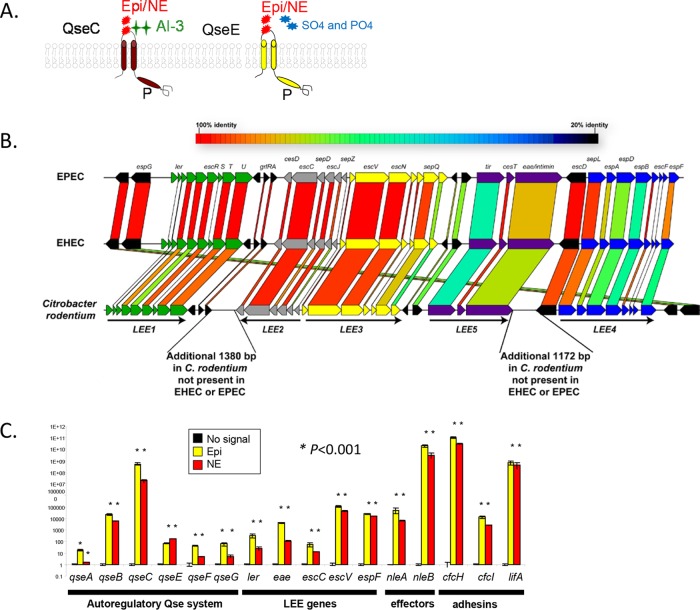
Virulence gene regulation in *C. rodentium* by epinephrine and norepinephrine. (A) Scheme of recognition by the QseC and QseE bacterial adrenergic receptors of their signals. QseC senses epinephrine (Epi) and norepinephrine (NE), and QseE senses Epi, NE, sulfate (SO_4_), and phosphate (PO_4_). (B) Homology comparisons among the LEE regions of EHEC, enteropathogenic *E. coli* (EPEC), and *C. rodentium*. (C) Expression of genes within the QseC regulon, the LEE, effectors, and adhesins in the absence and presence of 50 µM epinephrine or norepinephrine in DMEM (OD_600_ of 0.7 at 37°C).

One limitation of the infant rabbit model is the inability to generate knockout animals to assess the role of the host genetic repertoire in pathogen-host associations. To address this limitation, we employed the *C. rodentium* murine infection model. *C. rodentium* has the LEE genes (Fig. 3B) and colonizes and forms AE lesions in the colon (51, 52), which is the same GI site colonized by EHEC (58). Importantly, *C. rodentium* harbors the QseC and QseE adrenergic sensors. Both epinephrine and norepinephrine increased expression of virulence genes in *C. rodentium*, including the LEE genes (which encode a T3SS essential for intestinal colonization, colonic hyperplasia, and pathogenesis [52]), several effector genes (*nleA* and *nleB*), adhesin genes, and many genes within the QseC and QseE regulatory cascade (Fig. 3C; see also Fig. S2 in the supplemental material).

QseC and QseE are involved in LEE and *nleA* gene regulation in EHEC, with QseC activating their expression, while QseE represses it (24). Similarly to EHEC, QseC also activates expression of the LEE and *nleA* genes in *C. rodentium* (see Fig. S1 and S2 in the supplemental material). However, QseE has a contrasting function in *C. rodentium*; while it represses expression of the LEE and *nleA* genes in EHEC, it activates expression of these genes in *C. rodentium* (see Fig. S1 and S2). It is worth noting that, while QseC LEE and *nleA* gene regulation occurs directly by the activity of the QseC-phosphorylated response regulator KdpE in conjunction with Cra (59), activating expression of *ler* (59), which encodes the direct transcription activator of the LEE and *nleA* genes (29, 60), QseE regulation is indirectly controlled through the RcsB system (24). Moreover, QseE’s regulation also occurs posttranscriptionally, with QseE’s response regulator QseF activating expression of the GlmY small RNA (sRNA), which plays a role in the stability of the *LEE4* and *LEE5* operons and the *nleA* transcript (61, 62). Although the LEE is present in both EHEC and *C. rodentium*, several genes within the *LEE4* and *LEE5* operons have sequences that diverge between these two species (Fig. 3B), which could account for this differential regulation. Importantly, both QseC and QseE are involved in sensing epinephrine, with the *qseC* and *qseE* mutants being unable to activate the expression of the LEE and *nleA* genes in response to this signal (see Fig. S2 in the supplemental material).

**FIG 4:**
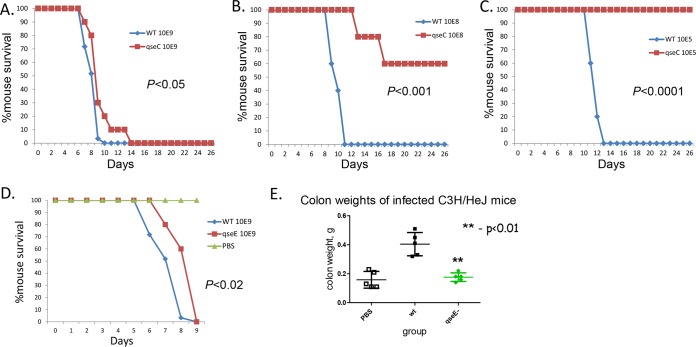
*C. rodentium* murine infections in C3H/HeJ mice. (A) Survival curves of animals infected with different doses of the WT strain and the *qseC* mutant with an infectious dose of 10^9^ CFU. (B) Survival curves of animals infected with different doses of the WT strain and the *qseC* mutant with an infectious dose of 10^8^ CFU. (C) Survival curves of animals infected with different doses of the WT strain and the *qseC* mutant with an infectious dose of 10^5^ CFU. (D) Survival curves of animals infected with the WT strain and the *qseE* mutant (*P* < 0.02). (E) Colon weights of animals infected with the WT strain and the *qseE* mutant; PBS is the negative control.

QseC and QseE are necessary for LEE gene activation in *C. rodentium* (see Fig. S1 and S2 in the supplemental material), and we have previously published results showing that *qseC*, *cra*, and *kdpE C. rodentium* mutants are attenuated for murine infection using high infectious doses of 10^9^ CFU (63, 64) and employing susceptible C3H-HeJ animals that succumb to death upon *C. rodentium* infection (65). Here we first refined these studies using different infectious doses to gain a full understanding of the attenuation of the *qseC* mutant. All experiments were performed with doses of 10^5^, 10^8^, and 10^9^ CFU using 10 mice per group (Fig. 4A to C). The WT-infected animals all succumbed to death eventually, with the lower infectious doses prolonging the time to death. The *qseC* mutant was attenuated compared to the WT results at all infectious doses, and at the lowest infectious dose (10^5^), all of the animals infected with the *qseC* mutant survived, while all animals infected with the WT strain succumbed to death (Fig. 4C). We also tested the *C. rodentium* qseE mutant for murine infection using an infectious dose of 10^9^ CFU. Death of Δ*qseE* mutant-infected animals was delayed by 1 day compared to the WT results, a difference which is statistically significant (*P* < 0.02) (Fig. 4D). The colon weights of Δ*qseE* mutant-infected animals (a readout of colonic hyperplasia) were lower than the weights measured for animals infected with the WT strain, being similar to those seen with the control animals administered phosphate-buffered saline (PBS) (Fig. 4E). Of note, we have previously published that the colon weight of Δ*qseC* mutant-infected animals was also lower than that of WT-infected animals (63). Taken together, these data indicate that both the *qseC* and *qseE C. rodentium* mutants are attenuated for murine infection, in accordance with the role of these two sensors in activating expression of the virulence genes (see Fig. S1 and S2).

### Epinephrine and norepinephrine are required for full virulence of *C. rodentium* during mammalian infection.

**FIG 5:**
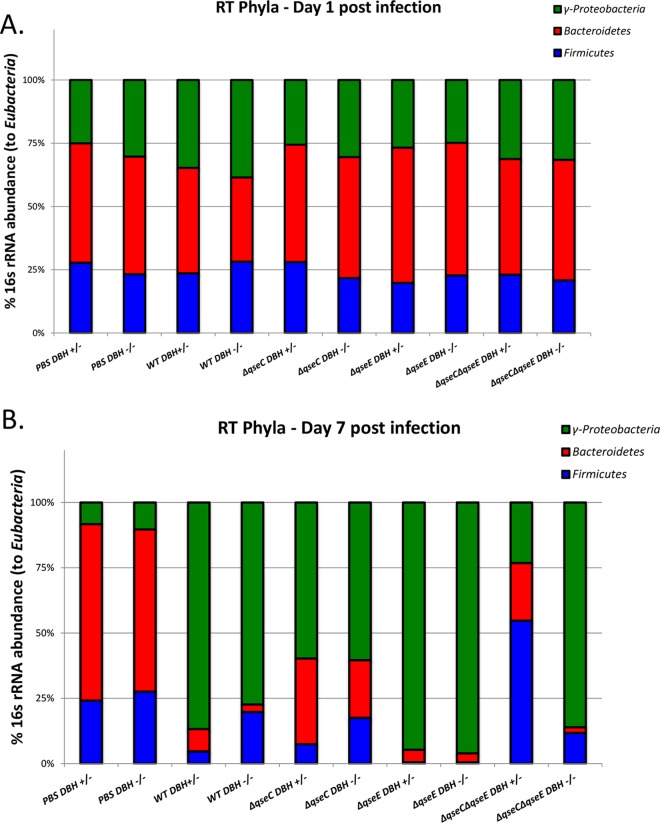
Microbiota composition of *Dbh^+/−^* and *Dbh^−/−^* mice infected with WT, Δ*qseC*, Δ*qseE*, and Δ*qseEC C. rodentium* at day 1 (A) and day 7 (B) postinfection. PBS mice were used as negative controls.

**FIG 6:**
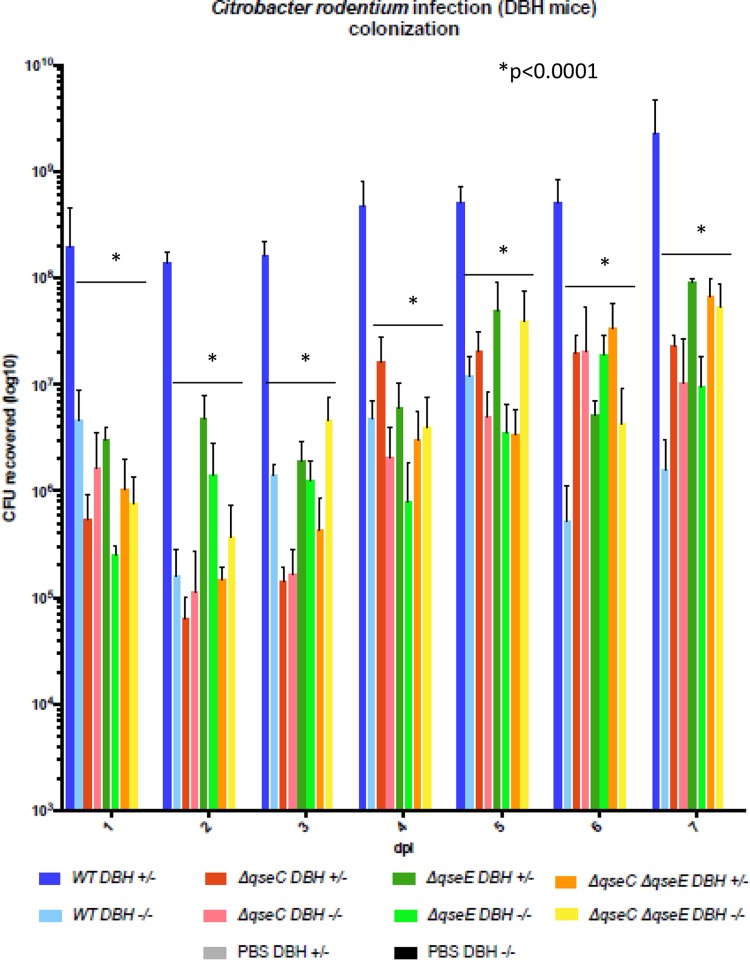
CFUs in stools of *Dbh^+/−^* and *Dbh^−/−^* mice infected with WT, Δ*qseC*, Δ*qseE*, and Δ*qseEC C. rodentium* on days 1 to 7. PBS mice were used as negative controls. *, *P* < 0.0001.

**FIG 7:**
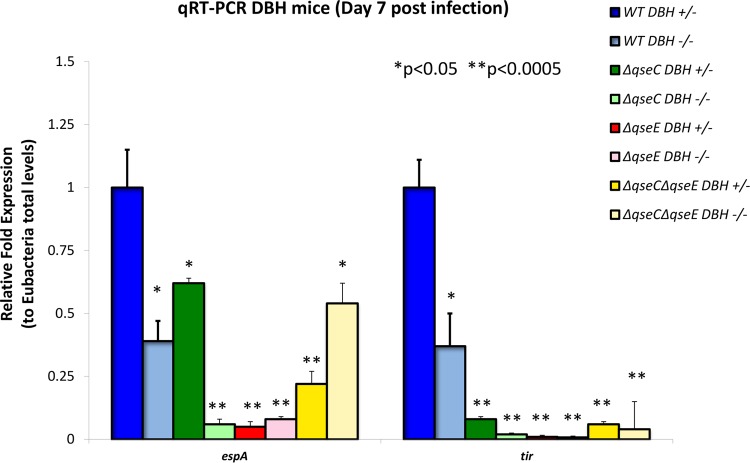
Expression of the LEE *espA* and *tir* genes, measured by qRT-PCR, in stools of Dbh^+/−^ and Dbh^−/−^ mice infected with WT, Δ*qseC*, Δ*qseE*, and Δ*qseEC C. rodentium* on day 7 postinfection. PBS mice were used as negative controls. *, *P* < 0.05; **, *P* < 0.0001.

Norepinephrine is synthesized from dopamine by the enzyme dopamine β-hydroxylase (Dbh), and phenylethanolamine-N-methyl transferase (PNMT) synthesizes epinephrine from norepinephrine. To investigate the role of epinephrine and norepinephrine in *C. rodentium* infection, we devised experiments using Dbh knockout animals (*Dbh*^−/−^). These experiments were performed using *Dbh*^+/−^ mice (the heterozygous mice are known to behave as WT for production of epinephrine and norepinephrine and represent the parent strain for the knockout animals), and the *Dbh*^−/−^ mice (which do not produce any epinephrine or norepinephrine) (66). However, these mouse strains are derived from a mix of C57BL6/J and 129x1/SvEv mice, which are more resistant to *C. rodentium* infection than C3H-HeJ mice. 129x1/SvJ mice are colonized at high numbers by *C. rodentium* and develop colonic hyperplasia and inflammation and compromised crypt integrity and also lose goblet cells, similarly to the results of *C. rodentium* infection of C3H-HeJ. However, unlike C3H-HeJ mice, 129x1/SvJ mice resolve and survive infection (65). Our pilot studies with 129x1/SvJ animals also confirmed that they develop disease and are colonized by *C. rodentium* but do not die (see Fig. S3 in the supplemental material). Hence, we decided not to perform survival studies in the *Dbh*^+/−^ and *Dbh*^−/−^ animals and to focus on other parameters of *C. rodentium*-mediated disease. In these resistant *C. rodentium* murine infection models, colonization of the intestine (CFU counts) correlates with the severity of the disease (65). These animals were infected with 1 × 10^9^ CFU of the *C. rodentium* WT strain and of the *qseC*, *qseE*, and *qseEC* mutants. We monitored CFU, LEE gene expression, and the composition of the microbiota in their stools during infection (Fig. 5, 6, and 7). We also performed comprehensive pathology analyses of their colons (Table 1). The animals were sacrificed on day 7, which, in this strain of mice, in our hands, corresponds to the peak of disease.

**TABLE 1:** Pathology scoring of large intestine

Sample	Score					
	Edema^a^	Crypt integrity^b^	Lymphocyte^c^	Neutrophil^d^	Apoptosis^e^	Vasculitis^f^
PBS *Dbh^+/−^*	0	0	0	0	0	0
WT *Dbh^+/−^*	2	2	3	1	4–8	3
Δ*qseC* *Dbh^+/−^*	0	0	1	0	0	0
Δ*qseE* *Dbh^+/−^*	0	0	4	0	1–2	1
Δ*qseEC* *Dbh^+/−^*	1	0	4–6	1	1–2	1
PBS *Dbh^−/−^*	0	0	4	0	0	0
WT *Dbh^−/−^*	3	4	6–10	4	6–8	1
Δ*qseC* *Dbh^−/−^*	1.5	2	5–6	6	5	1
Δ*qseE* *Dbh^+/−^*	0	2	5–6	4	2	0
Δ*qseEC* *Dbh^+/−^*	0	0	3	0	2	0

a Scores for (submucosal) edema are 0 for no edema and 5 for the highest edema in the submucosa.

b Scores for crypt integrity are 0 for normal crypts, 1 for irregular crypts, 2 for mild crypt loss, 3 for severe crypt loss, and 4 for complete crypt loss.

c Scores for lymphocytes represent the actual numbers of lymphocytes in the lamina propria (between adjacent crypts).

d Scores for neutrophilic infiltration in the wall (mainly perivascular in the submucosa but also seen occasionally in the lamina propria and the lumen) are 0 for no infiltration and 6 for the highest infiltration.

e Scores for apoptosis represent the number of apoptotic cells per ×600 field (five fields were counted). Numbers of apoptotic cells occasionally differed in areas of greater severity versus lesser severity; those instances are represented by two numbers.

f Scores for vasculitis are 0 for no evidence of vasculitis and 5 for the most severe vasculitis (i.e., loss of vessel wall architecture due to infiltration on leukocytes, presence of nuclear debris, and exudation of eosinophilic proteinaceous material; this is called leukocytoclastic vasculitis/fibrinoid necrosis in pathology literature). All of these changes are statistically significant (*P* < 0.05).

There are no previous reports on whether the presence or absence of epinephrine and norepinephrine affects the composition of the gut microbiota. Because the virulence and infectivity of EHEC and *C. rodentium* are altered by the microbiota (64, 67–69) and because the microbiota plays an important role in the availability of active epinephrine and/or norepinephrine in the lumen (5), we investigated the compositions of the microbiotas of uninfected and infected *Dbh*^+/−^ and *Dbh*^−/−^ animals at the phylum level. The compositions were similar on day 1 of *C. rodentium* infection (Fig. 5A). At day 7, the uninfected animals had microbiotas that were similar to those of each other and to their microbiotas prior to infection (Fig. 5B). Infection of *Dbh*^+/−^ and *Dbh*^−/−^ mice resulted in changes in the microbiota with all strains. However, these changes differed depending on whether these animals were or were not able to produce epinephrine and norepinephrine. At day 7, in animals infected with WT *C. rodentium*, the microbiota composition was dominated by γ-proteobacteria in both strains of mice, but the *Dbh*^−/−^ animals had more *Firmicutes* than the *Dbh*^+/−^ animals, and the *Dbh*^+/−^ animals had more *Bacteroidetes*. At day 7 postinfection, the Δ*qseC* mutant-infected animals also had their microbiota dominated by γ-proteobacteria in both strains of mice but to a lesser degree than the animals infected with the WT strain. The animals infected with the Δ*qseC* mutant showed an enhancement of *Bacteroidetes* and *Firmicutes* populations compared to WT-infected animals but kept the same pattern of the *Dbh*^+/−^ mice being enriched for *Bacteroidetes*, while the *Dbh*^−/−^ mice were enriched for *Firmicutes*. At day 7 postinfection, the Δ*qseE* mutant-infected animals had similar microbiota profiles in the *Dbh*^+/−^ and *Dbh*^−/−^ strains of mice. However, compared to WT-infected animals, Δ*qseE* mutant-infected animals had even more γ-proteobacteria and fewer *Bacteroidetes* and no *Firmicutes*. The most striking microbiota differences between *Dbh*^+/−^ and *Dbh*^−/−^ mice occurred at day 7 in animals infected with the double *qseEC* mutant. The microbiota of the Δ*qseEC* mutant-infected *Dbh*^+/−^ mice was dominated by *Firmicutes*, in contrast to animals infected with the WT strain, and presented the least prevalence of γ-proteobacteria. The microbiota of the Δ*qseEC* mutant-infected *Dbh*^−/−^ mice was very similar to the microbiota of the mice infected with the WT strain, with a prevalence of γ-proteobacteria and few *Bacteroidetes* and more *Firmicutes* in relation to *Bacteroidetes* (Fig. 5B). These studies suggest that the GI microbiotas are similar in the absence and presence of epinephrine and norepinephrine but that a *C. rodentium* infection has different impacts with respect to the changes that occur in the microbiota in the absence and presence of these hormones. Moreover, whether *C. rodentium* can sense these hormones also differentially impacts the composition of the microbiota in *Dbh*^+/−^ and *Dbh*^−/−^ animals. The observation that the microbiotas of *Dbh*^+/−^ and *Dbh*^−/−^ mice infected with the Δ*qseEC* mutant also differ suggests that there may be still another epinephrine/norepinephrine sensory mechanism in EHEC in addition to QseC and QseE. A third epinephrine/norepinephrine sensor has been suggested to exist in *Salmonella enterica* (70).

The levels of colonization differed dramatically when WT *C. rodentium* was used to infect *Dbh*^+/−^ versus *Dbh*^−/−^ mice. Throughout the infection, WT *C. rodentium* colonized *Dbh*^+/−^ animals to higher levels than the *Dbh*^−/−^ mice (differences of between 2 and 3 logs), suggesting that epinephrine and norepinephrine profoundly alter infection *in vivo* (Fig. 6). Colonization of the *qseC*, *qseE*, and *qseEC* mutants in the *Dbh*^+/−^ mice was decreased compared to the WT strain (Fig. 6), indicating that QseC and QseE promote colonization during infection. The difference in colonization of *Dbh*^+/−^ versus *Dbh*^−/−^ mice by any given strain was largely lost in the infections by the *qseC*, *qseE*, and *qseEC* mutants (Fig. 6), suggesting that differences in colonization upon infection of *Dbh*^+/−^ versus *Dbh*^−/−^ mice depend on both QseC and QseE, and indicating that both of these sensors are required to distinguish an intestine with epinephrine and norepinephrine from one without and to better colonize the former.

Expression of the LEE genes in WT *C. rodentium* was decreased in the *Dbh*^−/−^ animals compared to the *Dbh*^+/−^ animals, indicating that *C. rodentium* senses epinephrine and norepinephrine *in vivo* (Fig. 7). These data suggest that the absence of the host epinephrine and norepinephrine hormones in the *Dbh*^−/−^ mice prevents *C. rodentium* from optimally activating LEE gene expression, leading to decreased gut colonization (Fig. 6). Moreover, LEE expression is decreased in the *qseC*, *qseE*, and *qseEC* mutants compared to the WT strain during infection of both *Dbh*^+/−^ and *Dbh*^−/−^ mice, which suggests that the QseC and QseE sensors function *in vivo*. Expression of the LEE *espA* and *tir* genes was further decreased in the *Dbh*^−/−^ mice infected with the *qseC* mutant compared to infection of the *Dbh*^+/−^ mice and was decreased to similar levels in the two murine strains infected with the *qseE* mutant. Expression of *tir* was decreased in the *qseEC* mutant compared to the WT in both murine strains, but expression of *espA*, albeit reduced compared to the level seen with the WT in all animals infected with the *qseEC* mutant, was increased in the *Dbh*^−/−^ mice compared to the *Dbh^+/−^* animals (Fig. 7), which suggests that there is yet another manner to distinguish these animals in addition to analysis of QseC and QseE. Taken together, these data suggest that QseC and QseE are involved in *C. rodentium* sensing of epinephrine and norepinephrine in the gut to activate the LEE and colonize the GI tract.

Concerning intestinal pathology, uninfected *Dbh^−/−^* animals presented more lymphocytes than *Dbh^+/−^* animals, suggesting that epinephrine and norepinephrine play a role in preventing inflammation in the gut. Infection with WT *C. rodentium* led to increased edema, decreased crypt integrity, and increased inflammation in the *Dbh*^−/−^ animals compared to the *Dbh*^+/−^ animals. Although expression of the LEE and *C. rodentium* colonization were decreased during infection of the *Dbh*^−/−^ animals, the inherent increased inflammation in the intestine of these mice probably led to this increase in pathology, even though there was a smaller amount of pathogen, with decreased virulence. Infection with both the *qseC* and *qseE* mutants also led to increased pathology in the *Dbh*^−/−^ animals. However, infections with both mutants led to less pathology and inflammation than was seen with the WT in both the *Dbh^−/−^* and *Dbh^+/−^* mice (Table 1). These data suggest that although the activity of the *qseC* and *qseE* mutants was attenuated compared to that of the WT, the *Dbh*^−/−^ mice still presented more inflammation and decreased crypt integrity, even when infected with attenuated strains of *C. rodentium*, because of their inherent gut inflammation (66). The *qseEC* mutant, however, showed decreased pathology in comparison to the WT in both mouse strains and was similarly attenuated in the *Dbh*^+/−^ and *Dbh*^−/−^ mice. In fact, the pathology scores determined for the two mouse strains infected with the double mutant were largely similar to the scores determined for the uninfected (PBS-treated) animals. The *qseEC* mutant has both epinephrine/norepinephrine sensors deleted, suggesting that a *C. rodentium* strain that cannot sense these hormones is attenuated for disease and cannot even cause issues in the *Dbh*^−/−^ mice, even though they have inherent gut inflammation. It is worth considering that the QseC and QseE sensors also sense other signals in addition to epinephrine and norepinephrine. QseC senses microbiota-produced signal autoinducer-3 (AI-3) (19, 20), and QseE senses SO_4_ and PO_4_ (23). Hence, in addition to the absence of the adrenergic signals, the *qseEC* mutant is also impaired for sensing other signals that contribute to virulence gene regulation in EHEC and *C. rodentium*.

## DISCUSSION

Epinephrine and norepinephrine exert a profound effect in the host physiology and immune system, and the ability of bacteria to sense these hormones may facilitate gauging the fitness of the host (19, 20, 71). Specifically, in the GI tract, which is one of the most prominent sites in the human body where host/microbe associations are paramount, these neurotransmitters play important functions in gut homeostasis and physiology (19, 20, 71). This two-way-street communication is currently gaining appreciation given that there are important relationships between neurotransmitters and the GI microbiota, with the microbiota inducing biosynthesis of the serotonin neurotransmitter (72) and modulating the levels of active epinephrine and norepinephrine in the gut lumen (5). Disruption of these relationships and of the structure of the bacterial communities that inhabit the gut can contribute to dysbiosis leading to disease. An important insult that causes dysbiosis is infection by an invading pathogen. It is known that invading enteric pathogens such as *Salmonella enterica* and *C. rodentium* cause inflammation within the gut that in turn diminishes the overall numbers of bacteria in the microbiota, sometimes acting as a competition advantage to the pathogen (73, 74). Additionally, infection with *C. rodentium* also causes significant changes in the structure of the microbial community, decreasing the number of anaerobes and increasing the numbers of γ-proteobacteria (73). *C. rodentium* is a murine pathogen that models enteric infection by the human pathogen EHEC, and both pathogens sense epinephrine and norepinephrine to activate their virulence genes (11, 19–21, 23, 25, 26) (Fig. 3; see also Fig. S1 and S2 in the supplemental material).

These two neurotransmitters are sensed by the bacterial QseC and QseE sensors (20, 23). In both EHEC and *C. rodentium*, the *qseC* and *qseEC* mutants are attenuated for animal infections (7, 20, 63) (Fig. 2 to 4 and 6). The *qseEC* mutant is largely unable to detect and respond to epinephrine and/or norepinephrine (24) highlighting the important role of these neurotransmitters in enteric infection. However, the phenotypes of the *qseE* mutants differ between these pathogens (24) (Fig. 1 to 4 and 6; see also Fig. S1 and S2 in the supplemental material). An explanation for this difference is that QseE acts indirectly and posttranscriptionally in expression of the LEE island as well as of other effectors, such as NleA (24, 61). QseE exclusively phosphorylates its cognate response regulator (RR), QseF (75), while QseC acts through three RRs, its cognate QseB and the noncognates KdpE and QseF (21). The QseC tripartite signaling cascade activates LEE expression directly through KdpE and the gluconeogenesis sensor Cra (24). QseE acts indirectly through QseF, which directly activates expression of the *glmY* gene, and the GlmY sRNA mediates degradation of the *LEE4* and *LEE5* transcripts in EHEC (61). The homology between the *LEE1*, *LEE2*, and *LEE3* operons of EHEC and *C. rodentium* is high; however, the sequences of *LEE4* and *LEE5* are very divergent (Fig. 3B), and there is consequently a high probability that they would be differentially regulated at the posttranscriptional level. In fact, posttranscriptional regulation has been reported to differ even within EHEC strains (76–78). These data indicate that, although *C. rodentium* can be useful for addressing and mirroring certain aspects of EHEC pathogenesis, allowing for murine infections, one has to be cautious and aware of some of the limitations of this model. However, even with some of these limitations, the double *qseEC* mutant of both pathogens is attenuated for mammalian infection (Fig. 1 to 4 and 6).

The ability to harness the genetic tractability of murine models allowed us to investigate for the first time the role of epinephrine and/or norepinephrine impacting the pathogenesis of a noninvasive enteric pathogen. *C. rodentium*, as well as EHEC, does not breach the intestinal mucosa and invade the host systemically; the bacteria are restricted to the mucosal layer of the intestine (58, 79). It is very well established that norepinephrine is present in the basolateral layer of the intestine, where it is produced by adrenergic neurons of the enteric nervous system (2). However, whether it is present in the mucosal and luminal surface of the intestine, which is the compartment occupied by both EHEC and *C. rodentium*, was unresolved for many years. Recent evidence indicates that active norepinephrine is present in the lumen and mucosal layer and that the microbiota plays a key role in facilitating activation of this neurotransmitter (5). Using *Dbh^−/−^* knockout mice that do not produce epinephrine and norepinephrine, we have now been able to show that optimal LEE expression is dependent on these neurotransmitters and is also dependent on QseC and QseE (Fig. 7), leading to decreased levels of gut colonization by *C. rodentium* in these knockout animals (Fig. 6).

There is a growing appreciation of the complex microbial interactions within the gut-brain axis. Recent research has been elucidating how microbes can affect host behavior, and there is also a strong link between neurological function and the gut microbiota. Neurological diseases, such as autism, depression, and neurodegenerative diseases, are often strongly influenced by intestinal factors (80). The host neurotransmitters can also influence microbial behaviors, with studies of epinephrine and norepinephrine being at the forefront of this research (71). Although most of the epinephrine/norepinephrine modification of bacterial behavior was performed using gut pathogens, we could investigate these relationships *in vivo* only now using genetically tractable microbial and host systems.

## MATERIALS AND METHODS

### Isogenic mutant construction.

Nonpolar mutants of *qseE* and *qseEC* in *C. rodentium* were constructed through the use of a lambda Red system (81). Briefly, PCR products were amplified from plasmid pKD3 with flanking regions matching *qseE* and were transformed into the *C. rodentium* WT or the *qseC* mutant (63) expressing the RED genes from plasmid pKD46. After selection and confirmation, the resistance cassette was resolved using flippase from temperature-sensitive plasmid pCP20, which was then cured through growth at 37°C. This generated nonpolar mutants which were confirmed by sequencing.

### *In vitro* qRT-PCR.

Overnight cultures of WT (in the absence or presence of 50 µM epinephrine or norepinephrine) and Δ*qseC*, Δ*qseE* and Δ*qseEC* strains were diluted 1:100 and grown aerobically at 37°C in low-glucose Dulbecco’s modified Eagle medium (DMEM) (Gibco) to the exponential-growth phase (optical density at 600 nm [OD_600_] = 0.7). RNA was extracted from three biological samples using a RiboPure bacterial RNA isolation kit (Ambion) following the manufacturer’s guidelines. The primers used in the real-time assays were designed using Primer Express v1.5 (Applied Biosystems) (63) and were validated for amplification efficiency and template specificity. Quantitative real-time PCR (qRT-PCR) was performed as previously described (21) in a one-step reaction using an ABI 7500 sequence detection system (Applied Biosystems). Data were collected using ABI Sequence detection 1.2 software (Applied Biosystems).

All data were normalized to an *rpoA* (RNA polymerase subunit A) endogenous control and analyzed using the comparative cycle threshold (*C_T_*) method. Virulence gene expression was presented as fold changes over the WT expression level. Error bars indicate the standard deviations of the fold change values. The Student unpaired *t* test was used to determine statistical significance.

### Western blots of secreted proteins.

For Western blot analyses of secreted proteins, all cultures were grown in DMEM to an OD_600_ of 0.4 in the presence or absence of 50 µM epinephrine at 37°C, and proteins were isolated as previously described (31). Bovine serum albumin (BSA) (10 μg) was added to secreted proteins prior to concentration and loading such that the efficiencies of processing were known to be equivalent from sample to sample. Membranes were probed using an anti-EspB antiserum.

### WT mouse infection experiments.

For mouse survival experiments, 10 3.5-week-old female C3H/HeJ or 129x1/SvJ mice per group were infected by oral gavage with multiple infectious doses of wild-type and Δ*qseE* and Δ*qseC C. rodentium* strains by oral gavage with 100 µl of PBS. Mouse survival in each group (10 animals per group) was accessed over the course of 14 to 26 days. The Kaplan-Meier test was used to determine statistical significance.

For colon weight measurement, five 3.5-week-old female C3H/HeJ or 129x1/SvJ mice per group were infected with 1 × 10^9^ cells of wild-type and Δ*qseE* and Δ*qseC C. rodentium*. The infected mice were sacrificed on day 6 postinfection, and their colons were taken, washed, and weighed.

### *Dbh^−/−^* knockout mouse assays.

Dopamine β-hydroxylase knockout (*Dbh^−/−^*) mice, maintained with a mixed C57BL/6J and 129 SvEv background, were generated at Emory University as previously described (82) and were shipped to the University of Texas (UT) Southwestern Medical School. Next, these 129x1/Svj mice were housed in a specific-pathogen-free facility at the UT Southwestern Medical Center. All experiments were performed under IACUC-approved protocols. At 6 to 11 weeks of age, the mice were orally infected by gavage (either mock infected with PBS or orally infected with 1.3 × 10^9^ CFU of the *C. rodentium* WT strain [ICC168] or of Δ*qseC*, Δ*qseE*, or Δ*qseCE* isogenic mutants). Mice were monitored daily for survival. The experiments were performed at least twice with a total of 6 mice per group. Colonization changes and virulence gene expression levels were measured directly from RNA extracted from fecal pellets collected in triplicate daily up to day 7 postinfection. The mice were also monitored for weight changes and colon macroscopic differences after euthanization. CFU change curves reflect the averages and standard deviations (SD) of the experiment results. Littermate *Dbh^+/−^* heterozygous mice were considered controls because they had normal NE and Epi levels (83), as previously assayed with other enteric bacteria (8). The 2-way analysis-of-variance (ANOVA) multiple-comparison test was used to determine the statistical significance of the results of the comparisons of data determined each day and among the different mutants to WT levels.

### qRT-PCR for gene expression on murine stools.

The fecal pellets were collected from day 0 to day 7 after *C. rodentium* ICC168 infection of mice. RNA was extracted using a RiboPure Bacteria isolation kit according to the protocols of the manufacturer (Ambion). To assess host gene expression, tissue from the distal colon of infected mice was harvested on day 5 postinfection and homogenized in 1 ml TRIzol reagent (Life Technologies) per 100 mg feces or tissue. RNA was isolated using standard molecular biological procedures. The primers used for quantitative reverse transcription-PCR (qRT-PCR) of the LEE genes and the composition of the microbiota phyla (64) were validated for amplification efficiency and template specificity. qRT-PCR was performed as previously described (21) in a one-step reaction using an ABI 7500 sequence detection system (Applied Biosystems). Data were collected using ABI Sequence detection 1.2 software (Applied Biosystems).

All data were normalized to an endogenous control (*rpoA* for virulence gene expression in *C. rodentium*, *Eubacteria* 16S rRNA for total bacteria present in feces, or GAPDH [glyceraldehyde-3-phosphate dehydrogenase] for murine host gene expression in colonic tissue) and analyzed using the comparative cycle threshold (*C_T_*) method. Virulence gene expression was presented as fold changes in comparison to the expression level of WT *C. rodentium* cultured alone *in vitro* or *C. rodentium* (DBS770) infected alone *in vivo.* The relative abundances of *Bacteroidetes*, *Firmicutes*, and γ-proteobacteria (family *Enterobacteriaceae*) were measured by quantitative PCR (qPCR) with taxon-specific or universal 16S rRNA gene primers (64). The primers used were as follows: for *Eubacteria* (universal bacteria), Eub338F (5-ACTCCTACGGGAGGCAGCAGT-3) and Eub338R (5-ATTACCGCGGCTGCTGGC-3); for *Firmicutes*, 928F-Firm (5-TGAAACTYAAAGGAATTGACG-3) and 1040FirmR (5-ACCATGCACCACCTGTC-3); for *Bacteroidetes*, 798cfbF (5-CRAACAGGATTAGATACCCT-3) and cfb967R (5-GGTAAGGTTCCTCGCGTAT-3); and for γ-proteobacteria, 1080gF (5-TCGTCAGCTCGTGTYGTGA-3) and g1202R (5-CGTAAGGGCCATGATG-3) (64). Expression of each taxon was normalized to Eub388 expression and then compared to the expression level present in mock-infected (PBS) fecal pellets on day 0 (D0). Percentages of taxa were determined by dividing the value corresponding to expression of the taxon-specific 16S rRNA by the value corresponding to combined expression of *Firmicutes*, *Bacteroidetes*, and γ-proteobacteria. Student’s unpaired *t* test was used to determine statistical significance.

### Histopathology.

Portions of the distal colon and cecum were harvested 5 days postinfection with *C. rodentium*. The tissues were washed in PBS and then fixed in Bouin’s fixative for 48 h. The tissues were embedded in paraffin, cut into 5-µm sections, and stained with hematoxylin and eosin (H&E) in the UT Southwestern Pathology Core. Histological changes were analyzed in a double-blind fashion at Kansas State University. The severity of intestinal pathology was analyzed based on the following scoring system: for edema, 0 (no edema) to 5 (highest edema in the submucosa); for crypt integrity, 1, normal, 2, irregular crypts, 3, mild crypt loss, 4, severe crypt loss, and 5, complete crypt loss; for neutrophils, 0 for no infiltration to 6 for the highest neutrophilic infiltration in the wall; for apoptosis, 0 to 5 apoptotic cells per ×600 field (*n* = five fields); for bacterium attachment, 0 (no bacteria associated with the epithelial surface) to 5 (maximum number of bacteria associated with the epithelial surface); for goblet cells, average number of goblet cell in each crypt (*n* > 10 crypts); for vasculitis, 0 (no evidence of vasculitis) to 5 (the most severe vasculitis). The scores for each parameter represent averages of the cecum and distal colon results, taken from two independent experiments performed with 3 mice/experiment.

### EHEC infant rabbit experiments.

To prepare the inoculum, bacteria were grown overnight in LB broth at 37°C with appropriate antibiotics, harvested by centrifugation, and resuspended in sterile PBS (pH 7.2) and adjusted to a cell density of ~10^9^ CFU ml^−1^. Infant rabbit experiments were carried out as described previously (49). Briefly, 3-day-old New Zealand White rabbits were intragastrically inoculated with ~5 × 10^8^ CFU of WT EHEC or mutants using a size 5 French catheter. Rabbits were monitored twice daily for signs of illness or diarrhea. Diarrhea was described as (i) none—normal pellets were dark green, hard, and formed; (ii) mild—diarrhea consisting of a mix of soft yellow-green unformed and formed pellets resulting in light staining of the hind legs; or (iii) severe—diarrhea consisting of unformed or liquid feces, resulting in significant staining of the hind legs. Rabbits were euthanized at 2 and 5 days postinfection. At necropsy, the intestinal tract from the duodenum to the anus was removed and samples were obtained for microbiologic analyses. To limit any litter-specific effects, at least two different litters were used to test each bacterial strain.

## SUPPLEMENTAL MATERIAL

FIG S1  LEE gene expression in *C. rodentium*. (A) qRT-PCR of *ler*, *nleA*, *escV*, and *tir* in the WT, Δ*qseC*, and Δ*qseE* strains (in DMEM; OD_600_ of 0.7 at 37°C). (B) Western blot of EspB, from secreted proteins of WT Δ*qseC* and Δ*qseE C. rodentium*. BSA was used as a loading control. (C) qRT-PCR of *ler* and *escV* in the WT, Δ*qseC*, and Δ*qseE* strains and the complemented mutants (comp) (in DMEM; OD_600_ of 0.7 at 37°C). ***, *P* < 0.001, **, *P* < 0.01. Download Figure S1, PDF file, 0.1 MB

FIG S2  LEE gene expression in *C. rodentium* is activated by epinephrine in a QseC- and QseE-dependent manner. (A) Western blot of EspB, from secreted proteins of WT *C. rodentium*, in the absence and presence of 50 µM epinephrine. BSA was used as a loading control. (B) qRT-PCR of *nleA* in the WT and Δ*qseC* strains in the absence and presence of 50 µM epinephrine and in the complemented Δ*qseC* (Cpl) strain in the absence of epinephrine (in DMEM; OD_600_ of 0.7 at 37°C). (C) qRT-PCR of *escV* in the WT and Δ*qseC* strains in the absence and presence of 50 μM epinephrine (in DMEM; OD_600_ of 0.7 at 37°C). (D) qRT-PCR of *nleA* in the WT and Δ*qseE* strains in the absence and presence of 50 μM epinephrine (in DMEM; OD_600_ of 0.7 at 37°C). ***, *P* < 0.001. Download Figure S2, PDF file, 0.1 MB

FIG S3  *C. rodentium* infection of 129x1/SvJ mice. (A) Survival curves. (B) Colon weights at day 7 postinfection. (C) Gross pathology of colons at day 7 postinfection. Download Figure S3, PDF file, 0.1 MB
