# Supplementary material for: Bacterial Adrenergic Sensors Regulate Virulence of Enteric Pathogens in the Gut
Source: mBio. 2016 Jun 7;7(3):e00826-16. doi: 10.1128/mBio.00826-16 (PMC4959670; doi:10.1128/mBio.00826-16)
Supplement: FIG S1 — LEE gene expression in C. rodentium. (A) qRT-PCR of ler, nleA, escV, and tir in the WT, ΔqseC, and ΔqseE strains (in DMEM; OD600 of 0.7 at 37°C). (B) Western blot of EspB, from secreted proteins of WT ΔqseC and ΔqseE C. rodentium. BSA was used as a loading control. (C) qRT-PCR of ler and escV in the WT, ΔqseC, and ΔqseE strains and the complemented mutants (comp) (in DMEM; OD600 of 0.7 at 37°C). ***, P < 0.001, **, P < 0.01. Download [file mbo003162848sf1.pdf]

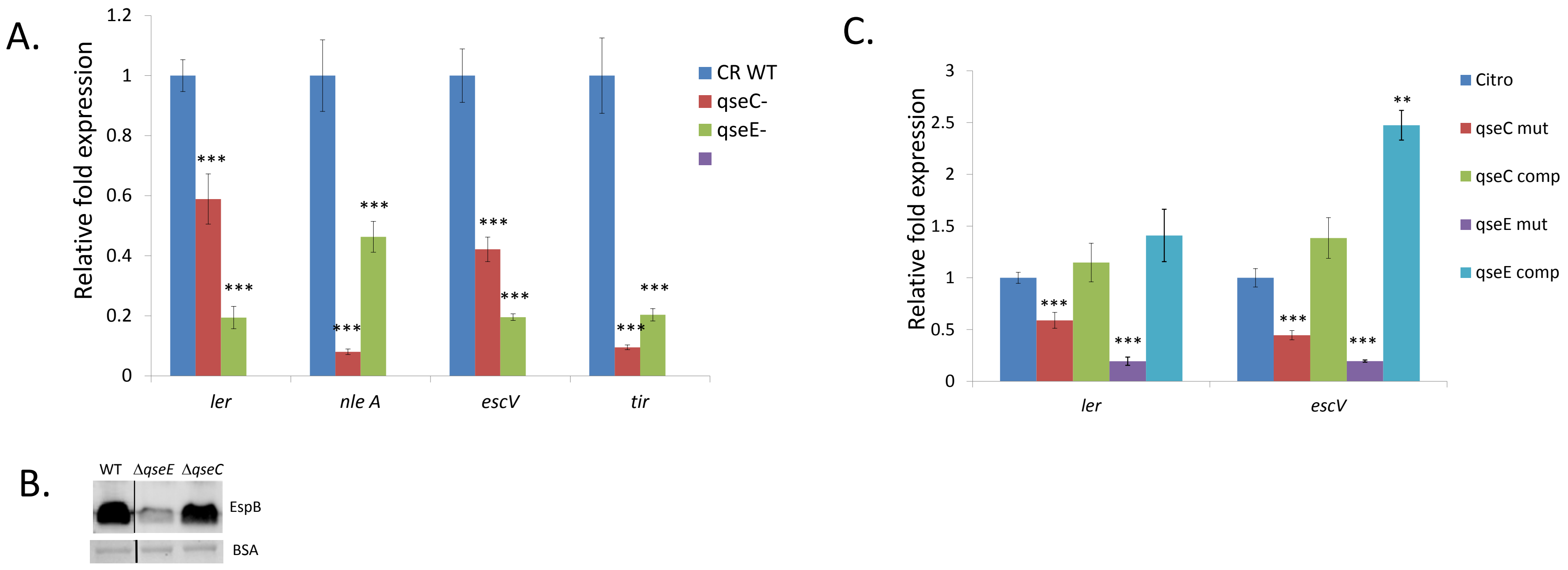

FIG S1. LEE gene expression in *C. rodentium*. (A) qRT-PCR of *ler, nleA, escV* and *tir* in WT, Δ*qseC* and Δ*qseE* in DMEM OD_{600} 0.7 at 37°C. (B) Western blot of EspB, from secreted proteins of WT Δ*qseC* and Δ*qseE C. rodentium*. BSA is a loading control. (C) qRT-PCR of *ler* and *escV* in WT, Δ*qseC* and Δ*qseE* and the complemented mutants (comp) in DMEM OD_{600} 0.7 at 37°C. *** $P < 0.001$; **$p<0.01$
