# Supplementary material for: Bacterial Adrenergic Sensors Regulate Virulence of Enteric Pathogens in the Gut
Source: mBio. 2016 Jun 7;7(3):e00826-16. doi: 10.1128/mBio.00826-16 (PMC4959670; doi:10.1128/mBio.00826-16)
Supplement: FIG S2 — LEE gene expression in C. rodentium is activated by epinephrine in a QseC- and QseE-dependent manner. (A) Western blot of EspB, from secreted proteins of WT C. rodentium, in the absence and presence of 50 µM epinephrine. BSA was used as a loading control. (B) qRT-PCR of nleA in the WT and ΔqseC strains in the absence and presence of 50 µM epinephrine and in the complemented ΔqseC (Cpl) strain in the absence of epinephrine (in DMEM; OD600 of 0.7 at 37°C). (C) qRT-PCR of escV in the WT and ΔqseC strains in the absence and presence of 50 μM epinephrine (in DMEM; OD600 of 0.7 at 37°C). (D) qRT-PCR of nleA in the WT and ΔqseE strains in the absence and presence of 50 μM epinephrine (in DMEM; OD600 of 0.7 at 37°C). ***, P < 0.001. Download [file mbo003162848sf2.pdf]

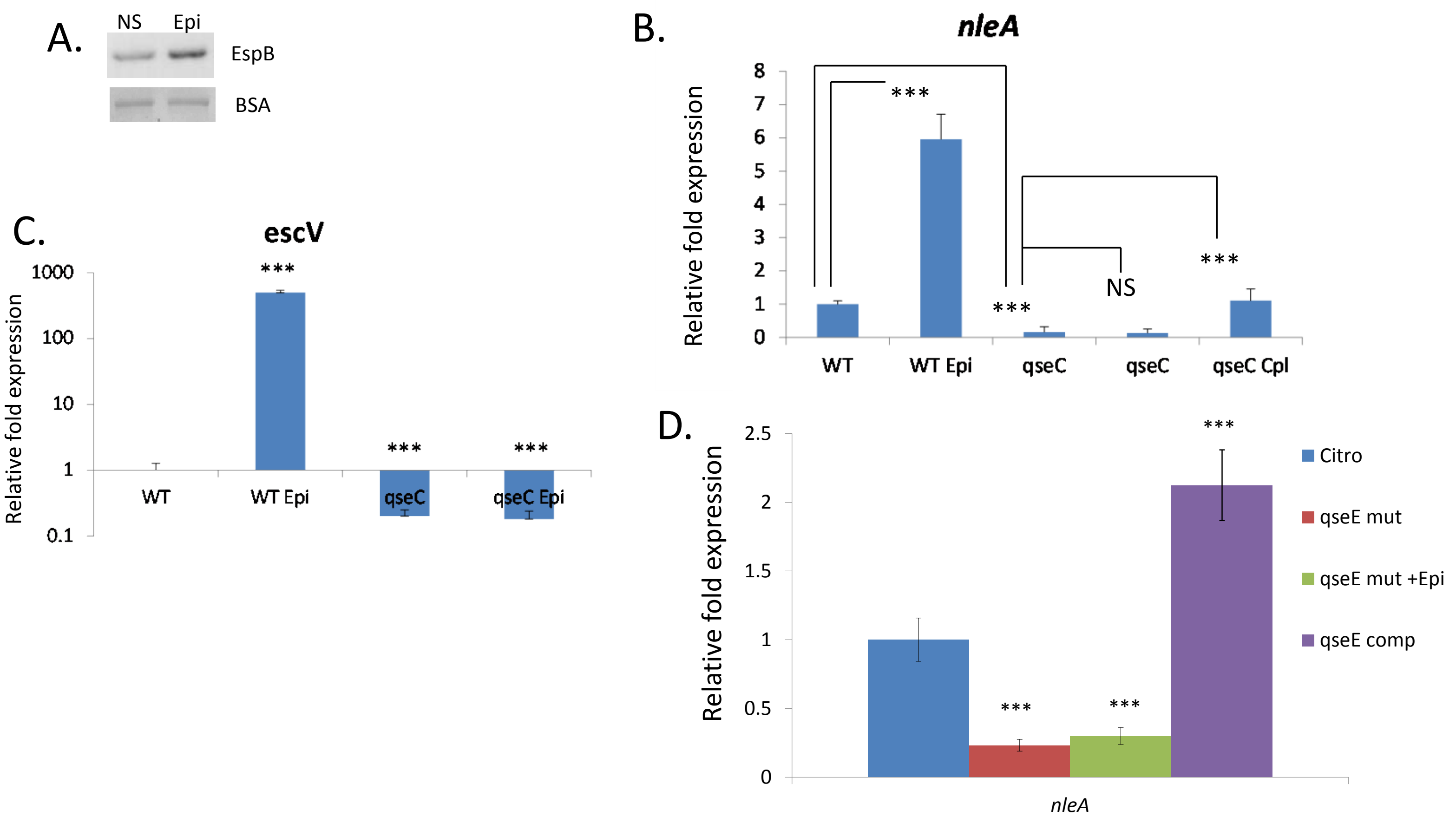

FIG S2. LEE gene expression in *C. rodentium* is activated by epinephrine in a QseC and QseE dependent manner. (A) Western blot of EspB, from secreted proteins of WT *C. rodentium* in the absence and presence of 50µM epinephrine. BSA is a loading control. (B) qRT-PCR of *nleA* in WT and Δ*qseC* in the absence and presence of 50µM epinephrine, and in the complemented Δ*qseC* (Cpl) in the absence of epinephrine in DMEM OD_{600} 0.7 at 37°C. (C) qRT-PCR of *escV* in WT and Δ*qseC* in the absence and presence of 50µM epinephrinein DMEM OD_{600} 0.7 at 37°C. (D) qRT-PCR of *nleA* in WT and Δ*qseE* in the absence and presence of 50µM epinephrinein DMEM OD_{600} 0.7 at 37°C. *** P<0.001
