# Supplementary material for: Bacterial Adrenergic Sensors Regulate Virulence of Enteric Pathogens in the Gut
Source: mBio. 2016 Jun 7;7(3):e00826-16. doi: 10.1128/mBio.00826-16 (PMC4959670; doi:10.1128/mBio.00826-16)
Supplement: FIG S3 — C. rodentium infection of 129x1/SvJ mice. (A) Survival curves. (B) Colon weights at day 7 postinfection. (C) Gross pathology of colons at day 7 postinfection. Download [file mbo003162848sf3.pdf]

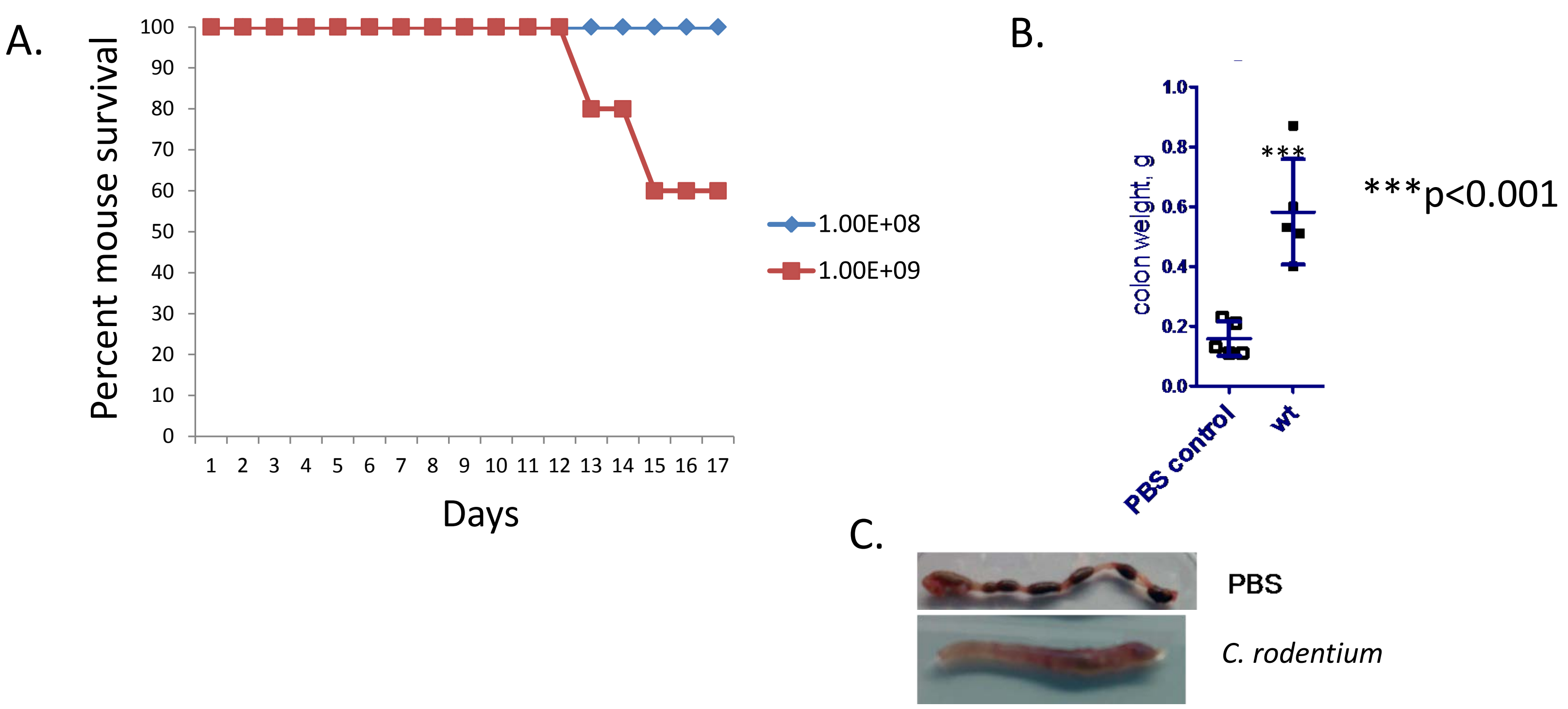

FIG S3. *C. rodentium* Infection of 129x1/SvJ mice. (A) Survival curves. (B) Colon weights day 7 post infection. (C) Gross pathology of colons at day 7 post infection.
